# Nutritional Management in Stricturing Crohn’s Disease: A Pilot Study

**DOI:** 10.3390/nu16234153

**Published:** 2024-11-29

**Authors:** Elisabetta Cavalcanti, Antonella Marra, Alessia Mileti, Rossella Donghia, Margherita Curlo, Mauro Mastronardi

**Affiliations:** 1 IBD Unit, National Institute of Gastroenterology-IRCCS, 70013 Castellana Grotte, Italy; antonella.marra@irccsdebellis.it (A.M.); alessia.mileti@irccsdebellis.it (A.M.); margherita.curlo@irccsdebellis.it (M.C.); mauro.mastronardi@irccsdebellis.it (M.M.); 2 Data Science Unit, National Institute of Gastroenterology-IRCCS, 70013 Castellana Grotte, Italy; rossella.donghia@irccsdebellis.it

**Keywords:** stricturing Crohn’s disease, EAA, sodium butyrate, nutrition, SMI index, body composition, BIA

## Abstract

Background: More than half of patients with Crohn’s disease develop intestinal fibrosis induced intestinal obstruction with debilitating symptoms throughout their disease course. The incidence of stricture formation in CD has remained unchanged over the last several decades. Factors promoting intestinal fibrosis are currently unclear, but diet may represent an underestimated risk factor for intestinal fibrosis by modification of both the host immune response and intestinal microbial composition. Evaluating the impact of diet on the course of IBD is very complex. Sarcopenia is a common problem in IBD patients and correlates with an increased rate of disease. Skeletal muscle index (SMI) is an important parameter to measure sarcopenia and is an easily accessible tool for evaluating the likelihood of complications in individuals with CD. Methods: Using a randomized and controlled pilot design, we aimed to investigate the efficacy of 12 months of short-term dietary intervention based on essential amino acid (EAA) and sodium butyrate (NaB) supplementation in the management of stricturing Crohn’s disease patients. Results: After the treatment in the diet EAA/NaB group, we revealed a statistically significant improvement of muscle mass (61.49 ± 5.47 vs. control 86 ± 10.70, *p* = 0.01) and SMI index (9.97 ± 1.79 vs. control 7.60 ± 2.29, *p* = 0.02). In addition, the measurement of skeletal muscle mass in CD patients has been suggested to be crucial for predicting the disease course. Indeed, after one year, surgery was required in 4/10 control group patients (40%) and 1/10 study group (10%) patients, underlining the importance of body composition alterations and adequate dietary intake in the management of these patients. Conclusions: Further prospective studies are needed to confirm these results; nonetheless this nutritional approach could become an integral part in the treatment of stricturing CD patients to improve disease outcomes and increase the quality of life in these patients.

## 1. Introduction

More than half of patients with Crohn’s disease (CD) develop intestinal obstruction caused by intestinal fibrosis and develop debilitating symptoms during the course of the disease. Inflammatory bowel disease-associated stricture has been seen as an irreversible process that frequently leads to intestinal obstructions requiring surgical intervention. The frequency of CD stricture formation has remained constant over the past few decades. Despite the increasing use of effective biologics and immunosuppressants, 80% of CD patients with intestinal fibrosis and strictures are resected, but postoperative recurrence and disease limitation are unfortunately common [1]. The lifetime risk of developing stenosis in patients with Crohn’s disease (CD) is approximately 50%. A significant increase in stricture frequency has been observed, which may indicate a chronicity of the disease rather than an improvement in the course of the disease in the era of biologic therapy. Although immunosuppression can reduce intestinal inflammation, it ultimately leads to a fibrotic and obstructive disease course due to stricture formation [2].

There is evidence that strictures in Crohn’s disease not only cause abdominal pain, bloating, gas, and vomiting, but may also precede the development of internal penetrating disease and form fistulas [3,4]. Stenosis consists of narrowing of the gastrointestinal tract and is a common disorder. A known long-term complication of CD is that 25% of patients with CD have at least one small bowel stricture and 10% of patients have at least one large bowel stricture [5]. Most strictures contain both inflammatory and fibrotic components, and the predominance of one over another informs optimal therapy [6]. Despite the unprecedented expansion of CD drug development over the past decade, novel and established treatments are primarily aimed at reducing inflammation [7]. Anti-inflammatory drugs may be effective in patients with small bowel strictures, but they do not specifically target or reverse fibrosis. Therefore, effective drug therapy to prevent and treat CD-related stenosis is a significant unmet medical need. This lack of progress may be due to multiple factors, including varying disease definitions, diagnostic methods, clinical trial eligibility criteria and endpoints, and treatment targets [8]. Factors promoting intestinal fibrosis are currently unclear, but diet may represent an underestimated risk factor for intestinal fibrosis by modification of both the host immune response and intestinal microbial composition. Evaluating the impact of diet on the course of IBD is very complex due to the elimination of certain foods and nutrients that may exacerbate symptoms or maintain disease remission. Chronic inflammation coupled with malabsorption and/or inadequate nutrient intake promotes the development of malnutrition in CD patients and affects their health and quality of life [9]. Nutritional status of patients with IBD is often compromised by imbalances in energy or nutrient intake manifested as malnutrition, including protein–energy malnutrition, disease-related malnutrition, sarcopenia, and micronutrient deficiencies. Malnutrition contributes to poor clinical outcomes in IBD patients, including increased episodes of disease, poorer response to medical therapy, higher rates of surgical complications, decreased quality of life, and prolonged hospital stays for patients hospitalized for IBD-related complications [10]. Previous studies demonstrated the prevalence and clinical significance of nutritional deficiencies in patients with IBD and highlighted the importance of screening for malnutrition early in the disease course to prevent disease-related complications [11]. Essential amino acids (EAA) may play a crucial role in the intestinal manifestations of disease as they are involved in many metabolic and immune functions [12,13]. Previous findings suggested that several AAs have potential therapeutic roles in intestinal diseases, particularly glutamine (Gln), the main fuel for intestinal epithelial cells, but current evidence still provides conflicting results [14]. Serum and tissue AA concentrations may vary depending on developmental stage, disease, nutritional status, endocrine status, and physical activity [15]. Variations in EAA concentrations were observed in IBD patients and in other different conditions such as liver diseases, heart failure, type 2 diabetes, and obesity [16]. In IBD patients, specific AA changes have been found in patients with active disease, suggesting that EAA profiles might be altered in this population and might reflect disease activity as well as patients’ nutritional status [17]. Many studies have examined the therapeutic potential of oral butyrate supplementation for intestinal inflammation in preclinical studies and clinical trials. Butyrate, a short-chain fatty acid, plays a crucial role in regulating intestinal immune function, maintaining barrier function, and intestinal homeostasis [18]. Butyrate was shown to reduce intestinal inflammation and relieve symptoms in a dose-dependent manner. Lee et al. reported that the daily oral supplementation of sodium butyrate at 100 mg/kg body weight reduced colitis scores, prevented body weight loss, and induced histone H3 acetylation in colonic mucosa in mouse models of acute and chronic colitis [19]. Moreover, butyrate treatment restored microbial community diversity and reduced microbiota dysbiosis in intestinal inflammation [20].

Using a randomized and controlled pilot design, we aimed to investigate the efficacy of 12 months of short-term dietary intervention based on the adoption of the diet with essential amino acid (EAA) supplementation and sodium butyrate (NaB) in the management of stricturing Crohn’s disease patients

## 2. Materials and Methods

### 2.1. Study Population and Clinical Data

A controlled pilot study was planned, and we enrolled 20 patients with fibrostricturing CD admitted to our IBD Unit between April 2022 and May 2023, assessed by MRI enterography at baseline. All participants were informed about the procedures and goals of this study. Patients gave written informed consent, and all procedures were carried out in correspondence with the 1964 Declaration of Helsinki and approved by the local Ethics Committee, “Comitato Etico I.R.C.C.S.-Istituto Tumori “Giovanni Paolo II”-Bari” (protocol code number 169 on 13 May 2022). The clinical features of the enrolled patients are shown in Table 1. Inclusion criteria were as follows: stricturing Crohn’s disease must have been diagnosed in the participants at least one/two years prior to the onset of the study, age of ≥18 years, and no history of abdominal surgery. Exclusion criteria were severe concomitant diseases, pregnancy, deliberate adherence to an extreme diet (e.g., macrobiotics, vegan), and celiac disease.

### 2.2. Assessment of Malnutrition Risk and Crohn’s Disease Activity Index

To evaluate the malnutrition risk, we assessed the malnutrition universal screening tool (MUST) according to the ESPEN guidelines [11]. MUST formulates a risk of malnutrition score based on current body mass index (0 points if BMI is >20 kg/m^2^; 1 if BMI is in the range of 18.5–20 kg/m^2^; 2 if BMI is <18.5 kg/m^2^), known weight loss (0 points if weight loss is <5%; 1 if between 5–10%; 2 if weight loss is >10%), and the presence of acute disease or no nutritional intake for a period equal to or greater than 5 days. The overall risk of malnutrition is determined from a total score ranging from 0 to 2, indicating the presence of a mild, moderate, or severe risk of malnutrition (low risk = 0, moderate risk = 1, and severe risk = 2) [21].

We evaluated the Crohn’s disease activity index (CDAI) scoring system to assess disease activity. CDAI was the most common index to assess the clinical status of CD patients, combining patient-reported signs and symptoms (loose/liquid stools, abdominal pain, general well-being) with clinical assessments (i.e., complications, presence of abdominal mass, change in weight, hematocrit levels, use of antidiarrheal agents), using a weighted scoring algorithm. Both the American College of Gastroenterology and the European Crohn’s and Colitis Organization define CDAI < 150, 250–220, 220–450, and >450 as reflecting remission, mild disease activity, moderate disease activity, and severe disease activity, respectively [22,23].

### 2.3. Nutritional Evaluation

Nutritional evaluation was performed by means of anthropometry and bioelectrical impedance vector analysis. Anthropometric measurements were taken by the principal researcher following standard criteria [24]. Height (cm) was measured with an anthropometer and weight (kg) with a mechanical beam scale; body mass index (BMI) (kg/m^2^) was hence calculated. Bioimpedance measurements were carried out by a trained investigator (IC) in subjects fasted for at least three hours. The patients were assessed by means of BIA from the beginning to the end of the follow-up with a portable, whole-body single frequency bioimpedance analyzer (Akern 101, Pontassieve, Itay) with the tetrapolar (4 electrodes) method. The BIA was carried out at enrollment and subsequently at the first check-up scheduled after 6 weeks and then every 14 weeks. The BIA-derived body composition parameters included body cell mass (BCM), body fat mass (FM), fat-free mass (FFM), phase angle (PhA), total skeletal muscle (MM), appendicular skeletal muscle mass (ASMM), and skeletal muscle index (SMI). Skeletal muscle index (SMI) was calculated as skeletal muscle (kg) adjusted for the squared height (kg/m^2^) as derived from BIA as per Janssen et al. [25]. The cut-off values, as recognized by the European Working Group on Sarcopenia in Older People (EWGSOP), were used to categorize patients in loss muscle volume. For males an SMI ≤ 8.75 kg/m^2^ and for females an SMI ≤ 5.75 kg/m^2^ were used to define the loss muscle volume [26].

The BIA was carried out at enrollment and subsequently at the first check-up scheduled after 6 weeks and then every 14 weeks. The following characteristics were collected from the patients’ outpatient medical records before starting the administration of the diet: age, sex, height, body weight (BW), body mass index (BMI), type of extension of the disease, localization stricture, stenosis thickness (cm), number of evacuations, abdominal pain, episodes of intestinal sub-occlusion, presence or absence of concomitant use of immunomodulators and corticosteroids, serum levels of C-reactive protein, and albumin. All patients were screened using the malnutrition universal screening tool (MUST). The time until bowel resection occurring was recorded from the medical records and was considered an indicator of long-term clinical outcome after dietary management. The diagnosis of sarcopenia included skeletal muscle mass and ASMM (appendicular muscle mass) measurement; BIA-derived SMI of <5.7 kg/m^2^ in women and <8.5 kg/m^2^ in men is considered severe sarcopenia.

### 2.4. Dietary Intervention

Individuals were divided into control and study group: (1) 10 patients underwent a normocaloric, high protein (1.2–1.5 g/kg/die), low fiber (10–15 g/die), and lactose-free diet; (2) 10 patients underwent a normocaloric, high protein, low fiber, and lactose-free diet with essential amino acid (EAA) and sodium butyrate (NaB) supplementation. Therefore, these 10 patients who received diet plus EAA/HBM were compared with the remaining 10 patients who received no diet supplementation. Depending on the level of undernutrition and the estimated requirements for each patient, a nutrition personalized plan was developed (usually with prescription of the normal energy intake for age and sex through consumption of varied foods following the Mediterranean diet pattern). Nutritional management was adjusted as needed based on the progress of the patient. In any case, patients were re-evaluated at regular intervals (every 6 weeks on an outpatient basis) to check adherence to the diet and correct any malnutrition. The diet of the control group includes a high-protein (to 1.2–1.5 g/kg/die), low fiber (10–15 g/die) and lactose-free Mediterranean diet. All diets were calculated using the METADIETA database (4.0.1. professional version; Meteda srl, Rome, Italy). The diet of the study group contained the same diet as the control group with essential amino acid (EAA) and sodium butyrate (NaB) supplementation. Ingestion of the supplement occurred twice daily, at 10 am and again at 5 pm. Supplement composition for the EAAs was as follows: leucine, 3.3 g (31.4% of total); lysine, 1.7 g (16.2%); isoleucine, 1.64 g (15.6%); valine, 1.64 g (15.6%); threonine, 0.92 g (8.8%); cysteine 0.39 g (3.7%); histidine, 0.39 g (3.7%); phenylalanine, 0.26 g (2.5%); methionine, 0.13 g (1.2%); tyrosine, 0.08 g(0.8%); and tryptophan 0.05 g (0.5%). Oral sodium butyrate (NaB) supplementation consisted of 2 tablets of 400 mg/day between meals, preferably in the morning and evening.

### 2.5. Statistical Analysis

Crohn’s patients’ characteristics are reported as mean and standard deviation (M ± SD) for continuous variables and as frequency and percentages (%) for categorical variables. To test the association between the independent groups (diet + ESS/NaB vs. control diet), the Fisher test was used for categorical variables, while the Wilcoxon Rank Mann–Whitney test was used to compare the two independent groups. A Friedman’s test was performed to evaluate changes of parameters reordered over follow-up (from first to last time).

The Spearman rank correlation coefficient was used to test the strength and direction of the association existing between the two examined variables (i.e., between SMI and BMI, PA, BCM, FM, MM, SMM, ASMM, and CRP, all evaluated as difference between the last and the first time).

In addition, the Wilcoxon matched-pairs signed-rank test for continuous variables was applied to evaluate variations between the 5th and 1st time of treatment for variables as CRP, CDAI, and SMI.

To test the null hypothesis of non-association, the two-tailed probability level was set at 0.05. The analyses were conducted with StataCorp. 2023. Stata Statistical Software: Release 18. College Station, TX, USA: StataCorp LLC., while RStudio (“Prairie Trillium” Release) was used for the plots.

## 3. Results

### 3.1. Baseline Characteristics of Patients

A total of 20 patients affected by scricturing CD were enrolled, 10 patients received EAA/NaB (study group), and 10 patients received no nutritional supplementation (control group). Randomization was used to eliminate bias between treatment groups. In fact, no significant difference was found in the baseline parameters as shown in Table 1. Age, gender, stenosis thickness, number of evacuations, and sub-occlusive episodes were not significantly different between the two groups at baseline. All patients were being treated with biological therapy. In the control group, 50% of patients were treated with infliximab (IFX), 10% with adalimumab (ADA) and 40% with ustekinumab (UST). In the study group, 30% of patients were treated with IFX, 60% with ADA, and 10% with UST. The dosage and frequency of administration of basic biological therapy were not modified in any group of patients.

Most CD patients present a disease ileocolic with stricture located at the ileocecal valve (VIC) (70% control vs. 90% study); a gastroduodenal disease with VIC was present in 20% control vs. 10% study group and only 10% in the study group presented a disease canal anal with VIC. The majority of patients observed (80% control vs. 70% study group) received at least one course of systemic steroids before starting biological therapy and none in the period of study. At baseline there were no differences in the CDAI scores of patient groups treated with medical therapies (*p* = 0.17; Table 1).

A total of 20 subjects were randomized to the normocaloric, high protein (1.2–1.5 g/kg/die), low fiber (10–15 g/die), and lactose-free diet group (*n* = 10) or the normocaloric, high protein, low fiber, and lactose-free diet with essential amino acid (EAA) and sodium butyrate (NaB) supplementation group (*n* = 10). Therefore, these 10 patients who received diet plus EAA/NaB were compared with the remaining 10 patients who received no diet supplementation. The primary endpoint was the ability of EAA/NaB dietary treatment to prevent sub-occlusive episodes at 1 year, preventing the patient undergoing surgery. The EAA/NaB diet had useful effects on multiple secondary endpoints, including body composition, SMI index and CDAI index leading to improved clinical outcomes and quality of life.

### 3.2. Nutritional Assessment

In this study, we first evaluated the risk of malnutrition-by-malnutrition universal screening tool (MUST) according to the ESPEN guidelines (10). The features of malnutrition were different in the control group versus the study group. The protective role of the diet on the MUST score was highlighted, in fact the relevance of patients that passed through the state of low risk of malnutrition was greater in the diet + EAA/NaB compared to the diet control group. Of note, at the beginning of treatment both the control diet groups showed 10% of patients with a low MUST score, 30% with a moderate score, and 60% with a severe score. Likewise, the diet EAA/NaB group presented 10% of patients with a mild MUST score, 50% with a moderate score, and 40% with a severe score. At the end of the treatment in the diet EAA/NaB group most patients (77.7%) had a mild MUST score compared to the control group, proving the effectiveness of the diet + EAA/NaB (Table 2).

### 3.3. Diet-Induced Changes in Body Composition Parameters and CDAI Index

The correct assessment of body composition was essential for an accurate diagnostic evaluation of nutritional status. Body composition can reflect the severity of disease activity status and disease behavior by characterizing the nutritional status of CD patients. The body mass index (BMI) was the most widely adopted indicator for evaluating undernutrition, overweight, and obesity, but it was unsuitable for differentiating changes in body composition.

In this study we analyzed BIA-derived body composition parameters on patients with stricturing Crohn’s disease treated to only diet and diet plus EAA/NaB. The diet EAA/NaB group showed a significant recovery of malnutrition and muscle function. Table 3 shows time-dependent treatment effects: recovery of BCM, PA, and MM were not observed in the control group. In particular, we highlighted that during the treatment the diet EAA/NaB and control group had a statistically significant difference in BCM (−1.85 ± 2.57 vs. 2.87 ± 2.01, *p* = 0.01), PA (−0.26 ± 0.75 vs. 0.77 ± 1.38, *p* = 0.003), and MM (−4.17 ± 3.40 vs. 2.73 ± 6.57, *p* = 0.01). The BMI value does not significantly change in the two groups despite the different distribution of FM, FFM, and MM. Analysis of body composition demonstrated a significantly decreased SMM and ASMM in the control compared to the diet EAA/NaB group.

In particular, we revealed a difference in ASMM between the study group vs. control group (2.27 ± 2.61 vs. 1.69 ± 2.85, *p* = 0.04) (Table 4). Table 4 demonstrates a significant relationship between the diet EAA/NaB and muscle mass overtime. After the treatment we revealed a statistically significant difference in SMI index between the study group vs. control group (9.97 ± 1.79 vs. 7.60 ± 2.29, *p* = 0.02).

**Figure 1:**
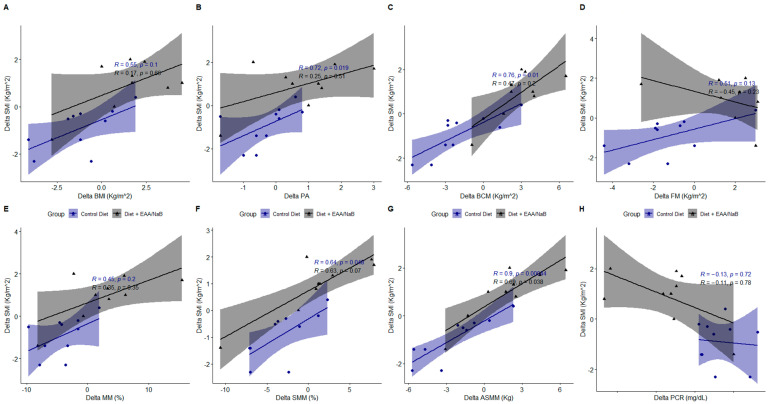
Correlations among the SMI index and body composition parameters, BMI (**A**), PA (**B**), BCM (**C**), FM (**D**), MM (**E**), SMM (**F**), ASMM (**G**) and CRP (**H**) assessed by BIA.

Correlation between SMI index and other parameters were evaluated. The parameters improved in both groups given that all patients followed a controlled dietary treatment. Statistical differences were found in the control group and not in the diet +EAA/NaB because diet + EAA/NaB improved all the parameters analyzed unlike the control group (Figure 1).

**Figure 2:**
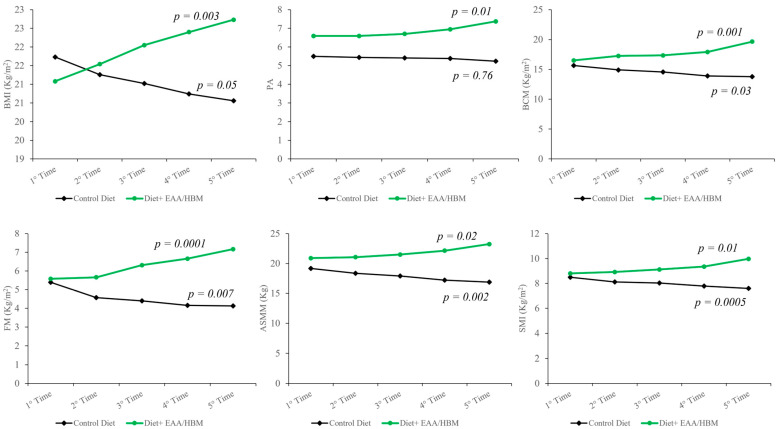
During the treatment time, diet + EAA/NaB brought about a shift in body composition with significant improvements in body mass index (BMI) (*p* < 0.003), phase angle (PA) (*p* < 0.01), body cellular mass (BCM) (*p* < 0.001), appendicular skeletal muscle mass (ASMM) (*p* < 0.02), and skeletal muscle index (SMI) (*p* < 0.01).

During the treatment time, diet + EAA/NaB brings a shift in body composition with significant improvement in BMI (*p* < 0.003), PA (*p* < 0.01), BCM (*p* < 0.001), ASMM (*p* < 0.02), and SMI (*p* < 0.01) (Figure 2).

Therefore, we evaluated how much the activity of the disease can be influenced by the diet. At the end of treatment in the diet control group there was no significant change in CDAI index (*p* = 0.34; Table 5). Although in the diet + EAA/NaB group, CDAI levels were significantly reduced (*p*. 0002). At the end of the treatment the average CDAI levels in the diet control group (327.00 ± 51.30) were elevated in comparison to the diet + EAA/NaB group (165.10 ± 60.92; *p* = 0.0001). Moreover, at the end of the treatment the average CRP levels in the diet control group (8.70 ± 3.35) were elevated in comparison to the diet + EAA/NaB group (2.97 ± 4.48; *p* = 0.003).

In addition, the measurement of skeletal muscle mass in CD patients, CRP, and CDAI levels have been suggested to be crucial for predicting the disease course. Indeed, after one year in this study, surgery was required in 4/10 control group patients (40%) and 1/10 study group (10%) patients, underlining the importance of body composition alterations in the management of these patients

## 4. Discussion

We believe this is the first report that has aimed to correlate diet with supplementation EAA/NaB, changes in body composition, CDAI index, and long-term outcomes (need for surgery as well as secondary failure) in stricturing CD patients. Very few studies have shown the relationship between the status of skeletal muscle and the clinical outcomes of CD patients. Zhang et al. reported the relationships between decreased skeletal muscle volume and postoperative complications [27]. However, the relationship between reductions in skeletal muscle volume and prognosis of IBD patients has yet to be elucidated. Low muscle mass and malnutrition may intersect throughout the health continuum and occur as a direct consequence of chronic disease or its treatment, as observed in Crohn’s disease. Body composition assessment was important for both clinical and research applications, particularly identifying patients with low muscle mass or muscle loss and assessing the effectiveness of dietary interventions. As a common measurement in clinical settings, BMI was not an indicator of muscle health and therefore was not a suitable indicator of body composition. Possible mechanisms of body composition changes in IBD were different. An interesting systematic review included 39 studies for analysis and reported a prevalence of sarcopenia > 50% in patients with CD [28]. Accelerated sarcopenia may happen in IBD patients related to inflammation, dietary restriction (insufficient intake of energy and nutrients), malabsorption, restrictive surgeries, reduced mobility, and malnutrition (specific and/or generalized). Malnutrition condition in patients with CD was characterized by reduced muscle mass and/or altered fat distribution [29]. Intestinal inflammatory response was a trigger for changes in body composition in CD patients. Body composition was affected by disease subtype, stage and nutritional status but not by lesion location [30]. In turn, quantitative monitoring of body composition changes in CD patients may help reflect the severity of inflammation (disease activity status and disease behavior) by characterizing the nutritional status of CD patients [31]. Nutrition was central to support muscle anabolism, reduce catabolism, and improve outcomes in patients with muscle loss and malnutrition. Assessment of nutritional status in IBD was the first step to identify malnutrition and/or sarcopenia status in order to set adequate and customized therapeutic regimens. Moreover, nutrition interventions were most beneficial when they were proactive, initiated early, and continued through recovery.

In this study we aimed to evaluate if an adequate dietary treatment could bring benefits to patients with inflammatory stenosis; however, there was currently no data to suggest that the diet had benefits. These patients were mostly malnourished because they had drastically reduced their food intake by limiting the consistency and especially the variety of foods; a special concern for patients with stricturing disease was that they may develop obstructive symptoms. Indeed, at the time of enrolment all patients had a MUST score between medium and high risk of malnutrition, moderate–severe disease activity index and changes in body composition (BCM, PhA, MM, ASMM, and SMI). Insufficient protein intake is a risk factor for developing sarcopenia, as protein supplementation improves muscle anabolism, thereby preventing muscle mass loss.

Although studies have shown no significant differences in protein intake between patients with IBD and healthy individuals, the supply of these nutrients may not be sufficient to meet the needs of patients with UC and CD due to their increased needs. According to ESPEN recommendations [11], exacerbations of IBD protein intake should be estimated at 1.2–1.5 g/kg body weight, so there are similarities with the recommendations for sarcopenia patients [26]. In this study, control and study group diet included a high-protein (1.2–1.5 g/kg/die), low fiber (10–15 g/die), and a lactose-free Mediterranean diet. Additionally, an oral EAA/NaB supplementation was added to the study group. Appropriate concentrations of butyrate play a supportive role in correcting immune disorders and colonic epithelial barrier function, thereby enhancing the effects of other medications. Clinical trials and animal studies have shown that oral butyrate supplementation reduces mucosal inflammation and improves barrier function in UC and CD [18]. In this study sodium butyrate (NaB) was included because during the course of IBD, stricturing CD patients follow a variety of diets, including low-fiber and elimination diets, such as milk-free, gluten-free, and easy-digestible diets, to relieve symptoms or for fear of their recurrence. The crucial factors necessary for proper short-chain fatty acid (SCFA) production are the consumption of dietary fiber every day and microbial balance associated with the presence of SCFA-producing bacteria in the intestinal microbiota. Among SCFAs, butyric acid has the best proven beneficial effect, whereas the importance of the other acids remains incompletely understood [32]. IBD is characterized by aberrant immune response and barrier dysfunction and is associated with a reduced number of butyrate-producing bacteria in the gut. Butyrate was found not only to provide energy to colonic epithelial cells but also to help maintain intestinal integrity and modulate immune responses. Many of the studies demonstrated the efficacy of oral butyrate supplements, butyrate enema, butyrogenic diet, and bacterial supplements in the treatment of IBD. Microencapsulated sodium butyrate (MSB) has been linked with regenerative and anti-inflammatory properties of the large bowel mucosa. Butyrate at appropriate concentrations helps to maintain intestinal barrier function and regulate the immune response in the gut. Butyrate administration is largely safe, though a few adverse effects have been noted [33]. Lin et al. [33] noticed that butyrate at excessive doses (more than 150 mmol/L) induced minimal mucosal damage in the colon and distal ileum in newborn rats. In addition, IBD patients are exposed to fluctuations in body weight during the course of the disease, which is associated with a disturbance of the intestinal microbiome [34,35]. In stricturing CD patients, elements like drug use and dietary changes (particularly lowering fiber intake) along with dysbiosis may contribute to a decrease in SCFA production. Therefore, the anti-inflammatory as well as the trophic effect of NaB could be beneficial for these patients.

EAAs are nutrients with anabolic properties that may increase muscle mass or attenuate muscle loss via the stimulation of muscle protein synthesis (MPS). EAAs may play critical roles in the intestinal manifestations of disease, due to their involvement in many metabolic and immune functions. Variations in AA concentrations were observed in patients with IBD [36] and also in different other conditions such as liver diseases, heart failure, type 2 diabetes. In IBD patients, specific EAAs alterations were found in active patients, suggesting that AA profiles might be altered in this population and might reflect disease activity as well as patients’ nutritional status [16,37,38]. In an interesting study, Cioffi I. et al. [17] highlighted that specific AAs varied according to disease activity and protein intake, adjusted to body weight and disease status in CD patients. Loss of amino acids across the intestinal lumen, in particular leucine, was associated with poor muscle biosynthesis [39]. In IBD patients, inflammation (acute and/or chronic) may lead to reduced physical activity characterized by periods of bed rest and the generation of proinflammatory cytokines play an important role in activating the muscle proteolysis machinery. Probably systemic inflammation contributes to muscle loss in patients with IBD. With regard to this point, persistent systemic levels of IL-6 in the blood were shown to be associated with accelerated muscle loss and the development of sarcopenia in the elderly [40]. Of interest, IL-6 works through a JAK/STAT signaling pathway, which is an important molecular target in IBD therapeutics, given its critical role in the upregulation of numerous inflammatory cytokines [41]. Other authors suggested that skeletal muscle is in fact an endocrine organ and therefore its metabolism is impacted by hormonal alterations, steroids, cytokines, and myokines [42]. The pathogenesis of sarcopenia in IBD is not well defined, but malabsorption, reduced protein intake, chronic inflammation, dysbiosis, decreased physical activity, medication effects, and hormone signaling are thought to play a role [41]. Indeed, decreases in MPS as a result of increased circulating oxidative and inflammatory factors are more responsible than muscle protein breakdown for the decreases in muscle mass during chronic disease. Therefore, nutritional interventions that reduce oxidation or inflammation in conjunction with higher protein intakes that overcome the anabolic resistance may enhance the MPS response to feeding and either increase muscle mass or attenuate loss. In several pre-clinical IBD studies some AA like glutamines (Glns) have been tested as therapeutic agents, to contribute toward keeping integrity/functionality of the intestine by modulating gut inflammation, with promising results [12,14,43]. Clinical studies documented that EAA supplementation was shown to reduce the rate of inflammation and nosocomial infections in elderly post-acute patients [44], in patients with severe brain injury, in dysphagic stroke patients, and in elderly subjects after surgery following hip fracture. In this study we highlighted a significant relationship between the diet EAA/NaB, CDAI index, and muscle mass overtime. After the treatment in the study group, we revealed a statistically significant improvement of muscle mass, SMI index, and CDAI index. In addition, the measurement of skeletal muscle mass in CD patients has been suggested to be crucial for predicting the disease course. Although in the diet EAA/NaB group, the CDAI index was statistically significantly improved, some studies underlined that CDAI index was nonspecific and had poor correlation with endoscopic findings of strictures [45,46]. Really, the severity of stenosis cannot be accurately assessed by the CDAI index because the score system to assess disease activity depends mainly on subjective sensations such as the degree of abdominal pain in patients, while pain thresholds vary from patient to patient, which may affect the accurate assessment of disease activity. Anyway, CDAI together with SMI index, CRP levels, and some parameters of body composition (BCM, PhA) can help monitor the outcome of these patients, reflecting the nutritional status of the body. Low values of these parameters usually indicate malnutrition, low immunity, tolerance to disease, and insufficient recovery ability after disease, which may require attention.

Indeed, after one year in this study, surgery was required in 4/10 control group patients (40%) and 1/10 study group (10%) patients, underlining the importance of body composition alterations in the management of these patients. The decrease in SMI index might be a negative prognostic factor and a reliable and largely available tool to stratify the risk of complications in stricturing CD patients. The relationship between sarcopenia and clinical outcomes was investigated in a number of studies [41,47,48]. A common problem was that a consistent definition of sarcopenia does not exist, in particular in CD patients, a reliable normal range and a cutoff value for SMI to define sarcopenia are lacking. Identifying sarcopenic patients has several potential benefits in terms of CD treatment management. Nutritional aspects in inflammatory bowel disease were particularly relevant as they could potently contribute to disease morbidity and mobility. It can be discussed that a poor nutritional status, selective malnutrition, or sarcopenia can be associated with poor clinical outcomes, response to therapy, and quality of life.

## 5. Conclusions

This randomized and controlled pilot study, despite a relatively small study population, suggests that adequate dietary intake combined with EAA/NaB supplementation may improve body composition, CDAI index, and consequently clinical outcome in stricturing CD patients.

Further prospective studies are needed to confirm these results; however, this nutritional approach could become an integral part in the treatment of stricturing CD patients to improve disease outcomes and increase the quality of life in patients with IBD.

## Figures and Tables

**Table 1:** Baseline characteristics.

Parameters *	Diet		*p* ^^^
	Control (*n* = 10)	Diet + EAA/HBM (*n* = 10)	
Age (yrs)	49.30 ± 12.27	39.90 ± 12.56	0.12
Gender (M) (%)	4 (40.00)	4 (40.00)	0.99 ^†^
Stenosis Thickness (cm)	8.95 ± 1.61	8.40 ± 1.26	0.51
Number Evacuations	6.60 ± 1.26	5.50 ± 2.46	0.18
Stool Consistency (%)			0.99 ^†^
Liquids	0 (0.00)	1 (10.00)	
Semi-Formed	4 (40.00)	4 (40.00)	
Semi-Liquid	6 (60.00)	5 (50.00)	
Blood Stool (%)			0.43 ^†^
0	8 (80.00)	6 (60.00)	
+	1 (10.00)	3 (30.00)	
++	1 (10.00)	0 (0.00)	
+++	0 (0.00)	1 (10.00)	
Mucus Stool (Yes) (%)	6 (60.00)	9 (90.00)	0.30 ^†^
Abdominal Pain (Yes) (%)	4 (40.00)	4 (40.00)	0.99 ^†^
Subocclusive Episodes	0.80 ± 0.79	1.50 ± 1.18	0.15
Resection (Yes) (%)	4 (40.00)	1 (10.00)	0.30
Therapy (%)			0.09
IFX	5 (50.00)	3 (30.00)	
ADA	1 (10.00)	6 (60.00)	
Stelara	4 (40.00)	1 (10.00)	
Steroids (Yes) (%)	8 (80.00)	7 (70.00)	0.99
CDAI	308.30 ± 38.58	272.50 ± 51.50	0.17
Stenosis Location (%)			0.58 ^†^
Ileo-Colic + VIC	7 (70.00)	9 (90.00)	
Gastroduodenal + VIC	2 (20.00)	1 (10.00)	
Anal Canal + VIC	1 (10.00)	0 (0.00)	

* As mean and standard deviation for continuous variables, and as frequency and percentage (%) for categorical; ^^^ Wilcoxon rank-sum test (Mann–Whitney); ^†^ Fisher exact test. Abbreviations: IFX, infliximab; ADA, adalimumab; VIC, ileocecal valve.

**Table 2:** MUST index evaluation by comparing outcomes between the two groups.

		Control Diet		Diet + EAA/NaB	
		Star Diet	End Diet	Star Diet	End Diet
Patient MUST Score n(%)	MUST Mild (0)	1 (10)	1 (10)	1 (10)	7 (77.78)
	MUST Moderate(1)	3 (30)	4 (40)	5 (50)	1 (11.1)
	MUST Severe (>2)	6 (60)	5 (50)	4 (40)	1 (11.1)

**Table 3:** Changes in body composition according to BIA (across the study) during timing between two groups.

Parameters *	Diet		*p* ^^^
	Control (*n* = 10)	Diet + EAA/HBM (*n* = 10)	
*BMI* (kg/m^2^)			
1° Time	21.76 ± 4.01	21.08 ± 2.30	0.90
2° Time	21.26 ± 3.56	21.54 ± 2.33	0.64
3° Time	21.02 ± 3.69	22.05 ± 2.71	0.59
4° Time	20.74 ± 3.79	22.40 ± 2.78	0.38
5° Time	20.56 ± 3.79	22.73 ± 3.53	0.23
*p-value*	0.05 ^ѱ^ *(Δ_5°−1°_ = −1.17 ± 1.85)*	0.003 ^ѱ^ *(Δ_5°−1°_ = 1.50 ± 2.12)*	
*PA*			
1° Time	5.50 ± 0.95	6.59 ± 1.24	0.04
2° Time	5.44 ± 0.95	6.59 ± 0.97	0.04
3° Time	5.41 ± 1.01	6.70 ± 0.96	0.02
4° Time	5.38 ± 1.02	6.95 ± 1.00	0.003
5° Time	5.24 ± 1.02	7.34 ± 1.25	0.003
*p-value*	0.76 ^ѱ^ *(Δ_5°−1°_ = −0.26 ± 0.75)*	0.01 ^ѱ^ *(Δ_5°−1°_ = 0.77 ± 1.38)*	
*BCM* (kg/m^2^)			
1° Time	15.64 ± 3.77	16.50 ± 2.75	0.43
2° Time	14.91 ± 3.94	17.25 ± 3.22	0.10
3° Time	14.57 ± 4.02	17.34 ± 3.59	0.06
4° Time	13.91 ± 4.47	17.91 ± 3.61	0.03
5° Time	13.79 ± 4.66	19.65 ± 3.82	0.01
*p-value*	0.03 ^ѱ^ *(Δ_5°−1°_ = −1.85 ± 2.57)*	0.001 ^ѱ^ *(Δ_5°−1°_ = 2.87 ± 2.01)*	
*FM* (kg/m^2^)			
1° Time	5.39 ± 4.06	5.58 ± 3.99	0.97
2° Time	4.57 ± 3.31	5.66 ± 3.85	0.54
3° Time	4.40 ± 3.04	6.31 ± 4.09	0.42
4° Time	4.16 ± 2.86	6.66 ± 3.72	0.12
5° Time	4.13 ± 2.96	7.17 ± 3.98	0.07
*p-value*	0.007 ^ѱ^ *(Δ_5°−1°_ = −1.26 ± 1.98)*	0.0001 ^ѱ^ *(Δ_5°−1°_ = 1.55 ± 1.71)*	
*MM (%)*			
1° Time	56.03 ± 8.93	58.54 ± 6.92	0.68
2° Time	54.81 ± 9.52	59.05 ± 6.11	0.40
3° Time	54.19 ± 9.87	59.04 ± 5.75	0.22
4° Time	52.71 ± 10.40	60.24 ± 5.67	0.03
5° Time	51.86 ± 10.70	61.49 ± 5.46	0.01
*p-value*	0.0001 ^ѱ^ *(Δ_5°−1°_ = −4.17 ± 3.40)*	0.03 ^ѱ^ *(Δ_5°−1°_ = 2.73 ± 6.57)*	

* As mean and standard deviation for continuous variables; ^^^ Wilcoxon rank-sum test (Mann–Whitney) for each time in groups; ^ѱ^ Friedman test for repeated measures. Abbreviations: BMI, Body Mass Index; PA, Phase Angle; BCM, Body Cellular Mass; FM, Fat Mass; MM, Muscle Mass.

**Table 4:** Effect of diet on SMI index.

Parameters *	Diet		*p* ^^^
	Control (*n* = 10)	Diet + EAA/NaB (*n* = 10)	
*SMM* (%)			
1° Time	41.31 ± 8.41	43.03 ± 7.64	0.79
2° Time	40.10 ± 9.16	43.13 ± 6.39	0.39
3° Time	39.83 ± 9.59	43.43 ± 6.84	0.35
4° Time	39.00 ± 9.85	44.15 ± 6.77	0.22
5° Time	38.16 ± 10.51	44.87 ± 6.77	0.11
*p-value*	0.01 ^ѱ^ *(Δ_5°−1°_ = −3.15 ± 3.33)*	0.10 ^ѱ^ *(Δ_5°−1°_ = 1.10 ± 5.43)*	
*ASMM* (kg)			
1° Time	19.16 ± 5.29	20.90 ± 5.05	0.43
2° Time	18.37 ± 5.35	21.07 ± 5.20	0.16
3° Time	17.90 ± 5.05	21.50 ± 5.64	0.12
4° Time	17.22 ± 5.64	22.15 ± 6.09	0.06
5° Time	16.89 ± 5.90	23.25 ± 7.02	0.04
*p-value*	0.002 ^ѱ^ *(Δ_5°−1°_ = −2.27 ± 2.61)*	0.02 ^ѱ^ *(Δ_5°−1°_ = 1.69 ± 2.85)*	
*SMI* (kg/m^2^)			
1° Time	8.50 ± 2.10	8.82 ± 1.43	0.42
2° Time	8.12 ± 2.17	8.92 ± 1.47	0.20
3° Time	8.03 ± 2.13	9.12 ± 1.75	0.16
4° Time	7.79 ± 2.32	9.35 ± 1.71	0.05
5° Time	7.60 ± 2.29	9.97 ± 1.79	0.02
*p-value*	0.0005 ^ѱ^ *(Δ_5°−1°_ = −0.90 ± 0.91)*	0.01 ^ѱ^ *(Δ_5°−1°_ = 0.92 ± 1.07)*	

* As mean and standard deviation for continuous variables; ^^^ Wilcoxon rank-sum test (Mann–Whitney) for each time in groups; ^ѱ^ Friedman test for repeated measures. Abbreviations: BMI, Body Mass Index; PA, Phase Angle; BCM, Body Cellular Mass; FM, Fat Mass; MM, Muscle Mass.

**Table 5:** Variations of CRP, CDAI, and SMI parameters, at first and last time of treatment.

Parameters *	Control Diet		*p* ^ѱ^	Diet + EAA/NaB		*p* ^ѱ^	*p* ^†^
	1° Time	5° Time		1° Time	5° Time		
CRP	5.46 ± 1.84	8.70 ± 3.35	0.002	6.59 ± 4.06	2.97 ± 4.48	0.04	0.003
CDAI	308.30 ± 38.58	327.00 ± 51.30	0.34	272.50 ± 51.50	165.10 ± 60.92	0.002	0.0001
SMI	8.50 ± 2.10	7.60 ± 2.29	0.02	8.82 ± 1.43	9.97 ± 1.79	0.07	0.02

* As mean and standard deviation (M ± SD); ^ѱ^ Wilcoxon matched-pairs signed-rank; ^†^ Wilcoxon rank-sum test (Mann–Whitney) between groups for parameters in the last time.

## Data Availability

All data generated or analyzed during this study are included in this published article.
